# Depicting Coupling Between Cortical Morphology and Functional Networks in Major Depressive Disorder

**DOI:** 10.1155/da/6885509

**Published:** 2025-04-27

**Authors:** Peng Wang, Li Lu, Jinghua Wang, Yang Xiao, Li Sun, Yuhong Zheng, Jie Sun, Jinhui Wang, Shao-Wei Xue

**Affiliations:** ^1^Center for Cognition and Brain Disorders/Department of Neurology, The Affiliated Hospital of Hangzhou Normal University, Hangzhou, Zhejiang, China; ^2^Peking University Sixth Hospital, Peking University, Beijing, China; ^3^Institute for Brain Research and Rehabilitation, South China Normal University, Guangzhou, Guangdong, China

**Keywords:** coupling, functional networks, major depressive disorder, morphological similarity, structural networks

## Abstract

An enduring mystery in neuroscience is the intricate interplay between brain anatomical structure and functional dynamics, particularly in the context of mental disorders such as major depressive disorder (MDD). A pivotal scientific question arises: How does the cortical morphology–function coupling (MFC) manifest in MDD, and what insights can this coupling provide into the clinical manifestations of the disorder? To tackle this question, we conducted a comprehensive analysis using high-resolution T1-weighted structural magnetic resonance imaging (MRI) and resting-state functional MRI (rs-fMRI) data from a cohort of 830 MDD patients and 853 healthy control (HC). By constructing morphological and functional networks based on cortical gray matter (GM) morphology and regional rs-fMRI time series correlations, respectively, we aimed to quantify MFC by assessing the spatial correspondence between these networks. Results revealed that MDD patients exhibited a spatial hierarchical pattern of MFC similar to HC, with variations in specific networks. Specifically, lower coupling was observed in the visual network (VIS) and sensorimotor network (SMN), while higher coupling was noted in the default mode network (DMN) and frontoparietal network (FPN). Notably, MDD patients demonstrated significantly increased MFC within the VIS, SMN, and dorsal attention network (DAN) compared to HC. Furthermore, altered MFC in the VIS correlated positively with depressive symptom severity. These findings contribute to our understanding of the potential clinical significance of MFC alterations in MDD.

## 1. Introduction

Major depressive disorder (MDD) is a severe psychiatric condition characterized by persistent and pervasive feelings of sadness or a profound loss of interest in activities, accompanied by a range of symptoms that significantly disrupt daily life [1]. Recent advances in magnetic resonance imaging (MRI) technology have greatly deepened our understanding of the neurological substrates underlying MDD, particularly through investigations of structural, functional, and network-level brain alterations that offer valuable insights into the pathophysiology of MDD.

Previous research has emphasized the intricate interplay between anatomical structure and functional dynamics, laying the groundwork for their integrative capabilities [2, 3]. Although anatomical structure constrains the brain's functional activities, it also partially determines them. In turn, functional activities reshape the underlying anatomical architecture through neuromodulation and neuroplasticity, enabling the brain to adapt and respond to both internal and external changes [4]. This bidirectional relationship constitutes the physiological foundation for exploring brain structure–function coupling. Notably, in healthy individuals, such coupling is not uniformly distributed across the cortex but follows a spatially organized gradient, suggesting a hierarchical organization of functional specialization. Specifically, primary sensory and motor areas, such as the somatosensory and visual cortices, exhibit stronger coupling. While transmodal association areas, including the prefrontal cortex and the default mode network (DMN), display weaker coupling [5, 6]. This spatial gradient likely reflects the multilevel specialization of cortical function, shaped by neurodevelopmental and evolutionary pressures [7]. These findings support that structure–function relationships in the brain are not binary but exist along a continuum, underscoring the importance of examining gradual transitions in structure–function coupling across different cortical regions.

The abnormal interplay between anatomical structure and functional dynamics is increasingly recognized as critical for understanding neuropsychiatric conditions. Emerging evidence suggests that structural and functional disruptions in these disorders are mutually reinforcing, contributing to their pathophysiology [8–10]. In fact, alterations in structure–function coupling have been identified as a promising neurobiomarker across multiple disorders. For instance, bipolar disorder is characterized by dysregulated coupling in emotion-regulation networks, such as the anterior cingulate cortex and limbic system, along with hyper-coupling in sensorimotor regions [11]. Likewise, in Alzheimer's disease (AD), progressive neurodegeneration disrupts the hierarchical organization of coupling in the DMN and the medial temporal lobe, leading to the decoupling of structural integrity and functional connectivity, which underlies memory deficits and cognitive decline [12]. These findings raise the possibility that similar abnormalities may also play a critical role in MDD. Patients with MDD may exhibit a disrupted hierarchical coupling pattern, reflecting broader pathological alterations in brain structure and function. Structural MRI studies have consistently reported gray matter (GM) volume reductions in crucial brain regions [13, 14], including the prefrontal cortex [15], insula [16], and hippocampus [17, 18] in individuals with MDD. These regions are implicated in emotional processing [16], cognitive control, decision-making, and memory [19, 20]. Such structural changes may impair the integrity of key neural circuits, contributing to both the onset and persistence of depressive symptoms. Concurrently, functional MRI (fMRI) findings reveal dysfunctional activation patterns in MDD, particularly involving the amygdala and ventromedial prefrontal cortex, both of which exacerbate emotional regulation difficulties [21–23]. These functional aberrations, coupled with disrupted and hyperconnected functional networks among key brain regions [24, 25], may reinforce the persistent depressive symptomatology observed in MDD, further exacerbating symptom severity. Together, these structural and functional disruptions highlight the complex, interdependent mechanisms underlying MDD pathophysiology.

However, structural connectivity has traditionally been inferred from diffusion-weighted imaging, which primarily focuses on white matter (WM) tracts. While this approach provides valuable information, it captures only one dimension of the complex brain connectivity landscape. Other critical aspects, such as structural connectivity based on morphology similarity, may provide additional insights into this intricate system. Evidence has shown that cortical regions connected within morphological similarity networks (MSNs) share similar cellular architecture, exhibit axonal connectivity, and demonstrate high levels of coexpression of genes specialized for neuronal functions, underscoring their biological relevance [26]. The concept of MSNs, which is based on cortical GM, challenges conventional perspectives concentrating on WM and introduces a novel framework for understanding the interplay between brain anatomical structure and functional dynamics. MSNs are characterized by high stability, reproducibility, and reliability, offering a complementary approach to studying brain connectivity [27]. Among morphological metrics related to GM, GM volume demonstrates greater sensitivity compared to cortical thickness and surface area in identifying structural alterations within the cortex that directly influence functional connectivity [28, 29]. Research indicates that GM volume is closely associated with the integrity of neural networks, especially in regions involved in emotional regulation and cognitive processing [30], which are often disrupted in MDD. Importantly, GM volume changes are more pronounced in MDD, suggesting a stronger structural dysfunction in these regions. Despite these advances, the precise neurobiological pathways linking regional cortical alterations to impaired neural network function and subsequent depressive symptoms, termed morphology–function coupling (MFC), remain unclear. Furthermore, it is yet to be determined whether MFC exhibits a hierarchical organization similar to the levels of functional specialization, necessitating further research to clarify their role in the pathogenesis of MDD.

In this study, we endeavor to characterize MFC patterns in MDD patients by constructing MSNs based on cortical GM volume and establishing functional connectivity networks (FCNs) through Pearson correlation analysis of regional time series. We hypothesize that both MDD patients and healthy control (HC) exhibit a hierarchical organization of MFC across the cortex, reflecting the inherent linkage between brain structure and function. Moreover, we propose that MDD patients will show significant deviations from HC in these MFC patterns, potentially revealing novel neural correlates of MDD and advancing our understanding of the disorder.

## 2. Material and Methods

### 2.1. Participants

The sample data in this study originated from a publicly accessible dataset contributed by the REST-meta-MDD project [24] and the DIRECT Consortium [31]. Participants underwent preliminary screening based on essential criteria, including diagnosis, sex, age, education level, and 17-item Hamilton Depression Rating Scale (HAMD) scores. The inclusion criteria were as follows: (1) age range between 18 and 65 years; (2) head motion, measured by mean framewise displacement (FD), less than 0.2 mm; (3) high-quality data and satisfactory spatial normalization confirmed through meticulous visual inspections; (4) comprehensive mask coverage without signal loss in any region of interest (ROI) as defined by the Harvard–Oxford brain atlas; and (5) availability of data from at least 10 participants per site. Additionally, MDD patients with HAMD scores below 7 were excluded. Ultimately, 830 MDD patients and 853 HC were included, all of whom were right-handed, as confirmed by the Edinburgh Handedness Inventory.

MDD diagnosis was based on the Diagnostic and Statistical Manual of Mental Disorders, Fourth Edition (DSM-IV), or the International Classification of Diseases, Tenth Edition (ICD-10). Depression severity was measured using the HAMD, which includes nine items rated on a 0–4 scale and eight on a 0–2 scale, with a total score ranging from 0 to 52 (higher scores indicate greater severity). The assessed symptoms are (1) depressed mood; (2) guilt; (3) suicide; (4) insomnia, initial; (5) insomnia, middle; (6) insomnia, delayed; (7) work and interests; (8) retardation; (9) agitation; (10) anxiety, psychic; (11) anxiety, somatic; (12) somatic, gastrointestinal; (13) somatic general; (14) somatic genital; (15) hypochondriasis; (16) insight; (17) loss of weight. The research procedures adhered strictly to the ethical principles outlined in the Declaration of Helsinki and received approval from the Institutional Review Boards of all participating sites. Prior to engaging in the formal testing at their respective institutions, written informed consent was obtained from each participant.  Table 1 presents a comprehensive summary of the demographic and clinical characteristics of the study participants.

### 2.2. Image Preprocessing

The preprocessing of T1-weighted structural MRI and resting-state fMRI (rs-fMRI) data adhered to standardized pipelines tailored from the DPARSF software [32]. Initially, the T1-weighted images underwent segmentation into GM, WM, and cerebrospinal fluid (CSF) in their native space. Subsequently, the GM images were spatially normalized using the DARTEL toolbox [33] and then modulated by scaling them with the Jacobian determinants derived from the normalization process. Finally, the normalized GM volume was smoothed using a three-dimensional Gaussian filter with a full width at half maximum (FWHM) of 6 mm to mitigate spatial noise and enhance signal homogeneity.

For rs-fMRI data preprocessing, the initial 10 functional volumes were discarded to facilitate signal stabilization and mitigate potential environmental influences. Following this, slice timing correction was applied to compensate for acquisition delays across slices. Head motion realignment was then performed to correct for any motion artifacts during the scan. Subsequently, spatial normalization was executed to align the functional images to a standard anatomical template. Bandpass temporal filtering (0.01–0.1 Hz) was applied to retain neural activity fluctuations of interest while removing higher and lower frequency noise. Furthermore, Friston-24 motion parameters, along with WM and CSF signals, were regressed out as nuisance covariates to minimize their confounding effects on the follow-up analysis [34].

### 2.3. Constructing Morphological and Functional Brain Networks

The cerebral cortex was segmented into 96 distinct brain regions utilizing the Harvard–Oxford brain atlas as a reference, with each region serving as a node within a network framework. For each participant, the GM volume was extracted for each voxel within these segmented regions. The probability density function (PDF) of these GM volumes was estimated using kernel density estimation (KDE), with bandwidths automatically selected to ensure optimal fitting of the data. To quantify morphological connectivity between brain regions, the Kullback–Leibler divergence (KLD)-based similarity (KLS) method was employed. This approach resulted in the generation of a 96 × 96 KLS-based MSN for each individual participant, representating the morphological relationships between brain regions. The KLS is computed as follows:

  $$\begin{array}{c} \text{KLS}\left(P,Q\right)=e^{-D_{kl}\left(P,Q\right)}, \end{array}$$

where *e* is natural exponential. Through this transformation, the resulting KLS value ranges from 0 to 1, where 0 indicates that the GM density distributions of *P* and *Q* are maximally separable, whereas 1 indicates that the two distributions are identical. The KL distance is defined as follows:

  $$\begin{array}{c} D_{\text{KL}}\left(P,Q\right)=\sum_{i=1}^{n}\left(P\left(i\right)\log\frac{P\left(i\right)}{Q\left(i\right)}+Q\left(i\right)\log\frac{Q\left(i\right)}{P\left(i\right)}\right), \end{array}$$

where *n* is the total number of sample points. In this study, a total of 2^7^ sample points were utilized to estimate the parameters *P* and *Q* through KDE, adhering to methodologies established in previous research [35]. The optimal bandwidth for KDE was automatically determined employing the previous approach [36], which was implemented using the Botev code (function: kde, accessible at http://www.mathworks.com/matlabcentral/fileexchange/14034-kernel-density-estimator). Meanwhile, a 96 × 96 functional brain network was constructed for each participant by calculating Pearson's correlation coefficients between the mean rs-fMRI time series across all possible region pairs, reflecting the functional interactions between these regions.

### 2.4. Measuring Coupling Between Cortical GM Morphological and Functional Networks

**Figure 1:**
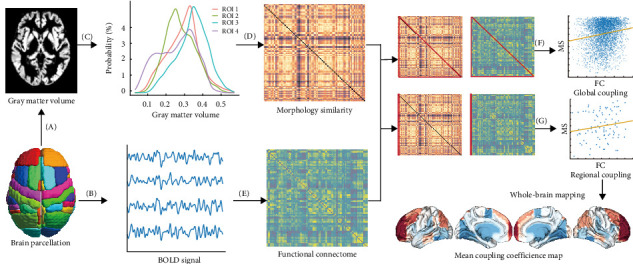
Schematic summary of the study design. The Harvard–Oxford brain atlas was used to parcellate the cerebral cortex into 96 regions, excluding the subcortical nuclei. (A) Gray matter volume for each voxel within the parcellated regions was extracted. (B) The average BOLD time series was obtained for each region. (C) KDE was applied to compute the probability density distribution of GM volume across different brain regions. (D) MSN was constructed based on the similarity of gray matter volume distribution. (E) FCN was computed by correlating the average BOLD time series between pairs of regions. (F) Global MFC was quantified by Spearman's rank correlation coefficient between the MSN and FCN matrices. (G) For each individual, regional coupling was computed by correlating the columns of the MSN and FCN matrices corresponding to each region. FCN, functional connectivity network; GM, gray matter; KDE, kernel density estimation; MFC, morphology–function coupling; MSN, morphological similarity network.

Figure 1 shows a conceptual overview for assessing regional MFC between the MSNs derived from individual GM morphology and the corresponding FCNs derived from rs-fMRI. Both rs-fMRI and GM segmentation data were spatially normalized to the Harvard–Oxford brain atlas, covering 96 distinct cerebral regions. This standardization ensures that the functional connectivity matrices have a common dimensionality with the morphological similarity matrices, facilitating direct comparisons. To quantify the relationship between morphological and functional networks, Spearman's rank correlation coefficient was computed pairwise between the columns of the morphological similarity matrix and the corresponding columns of the functional connectivity matrix. This approach measures the regional MFC strength for each subject, excluding self-connections within each column to focus on inter-regional associations. As a result, a 96-dimensional vector was generated for each participant, with each element representing the MFC value for a specific region's interactions with all other regions across the cerebral cortex. These region-specific MFC indices were then averaged across the cohort to produce a mean regional coupling map, providing a population-level representation of coupling patterns. A similar methodology was used to calculate global coupling indices, offering an overall measure of morphology–function coherence across the entire brain. Before conducting the regional MFC analysis, the ComBat algorithm was applied to mitigate the effects of multisite variability, ensuring that data harmonization corrected for site-specific biases and improved the robustness of subsequent analyses (https://github.com/Jfortin1/neuroCombat).

### 2.5. Associations of MFC Alterations in MDD With Meta-Analytic Cognitive Terms

To examine the correlation between MDD-associated brain changes in MFC patterns and meta-analytically derived cognitive constructs, we employed a Neurosynth-informed meta-analysis decoding approach (https://neurosynth.org/). Specifically, we used the “decoder” functionality within Neurosynth to quantify the spatial correlation between the MFC changes and meta-analytic maps corresponding to individual cognitive terms in the database. To ensure the robustness and validity of our findings, we conducted permutation testing to assess the significance of these correlation coefficients, adjusting for spatial autocorrelation. This approach allowed us to identify potential cognitive processes underlying the observed MFC alterations in MDD patients.

### 2.6. Statistical Analysis

A two-sample *t*-test was used to compare demographic differences (including age, education level, and head movement) between MDD participants and HC, whereas a chi-square test was employed to assess gender differences between the two groups. To examine differences in MFC patterns between MDD and HC groups, we applied a generalized linear model (GLM) to incorporate age, gender, head movement, and educational level as covariates in the regression analysis. Following this, Mann–Whitney *U* tests were conducted to assess group differences in coupling at the global, regional, and network levels.

To further explore MFC variations in relation to functional specialization, we calculated the mean MFC for each region across all subjects within the seven cortical networks defined by Yeo et al. [37] and applied a GLM to identify case–control differences, adjusting for the same covariates. Multiple comparisons were corrected using the Benjamini–Hochberg false discovery rate (BH-FDR) method, with a threshold set at *p* < 0.05. Furthermore, to elucidate the spatial organization of MFC, we examined its continuous spatial variation across cortical regions. Rather than computing a discrete gradient, we inferred hierarchical distribution patterns based on previous findings [5, 38], providing a framework for interpreting the coupling differences observed between MDD and HC.

The clinical relevance of MFC alterations was explored through partial correlation analyses, with adjustments for the same covariates to examine associations between significantly altered MFC networks and symptom severity (HAMD scores) in MDD patients. For these partial correlation analyses, a significance threshold of *p* < 0.05 was applied, with adjustments for multiple comparisons made using the BH-FDR method.

## 3. Results

### 3.1. Profiling the Coupling Relationships Between Cortical GM Morphological and Functional Networks

**Figure 2:**
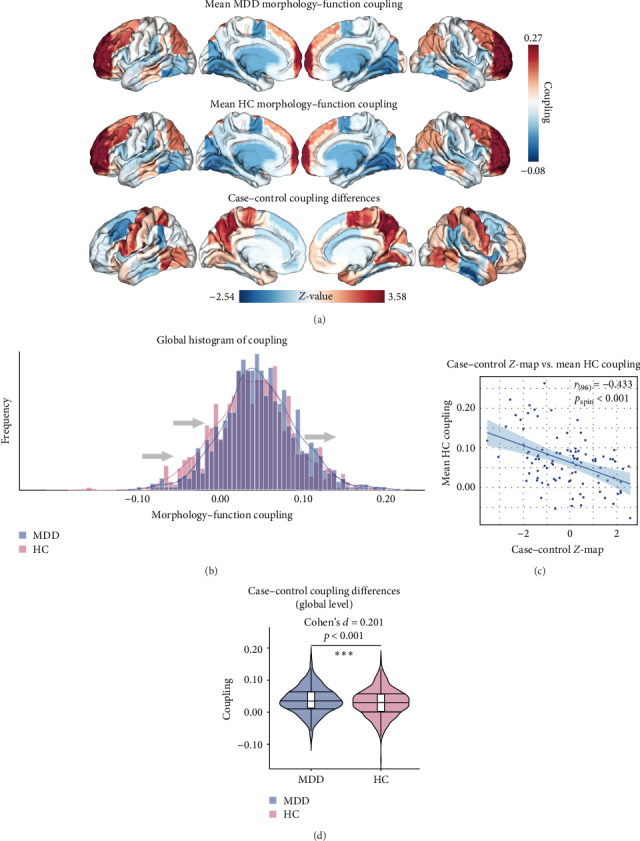
Coupling relationships between cortical gray matter morphological and functional networks. (A) Spatial distribution of global MFC in the MDD and HC groups, along with between-group differences. (B) Histogram depicting the global MFC distribution in MDD and HC, showing a rightward shift in MFC among MDD patients. (C) Density scatterplot illustrating the case–control *z*-value (*x*-axis) and the mean HC coupling (*y*-axis) (*r* = − 0.433, *p*_spin_ < 0.001). (D) Between-group statistical comparison of global MFC values in MDD and HC. *⁣*^*∗∗∗*^*p* ≤ 0.001, BH-FDR corrected. BH-FDR, Benjamini–Hochberg false discovery rate; HC, healthy control; MDD, major depressive disorder; MFC, morphology–function coupling.

**Figure 3:**
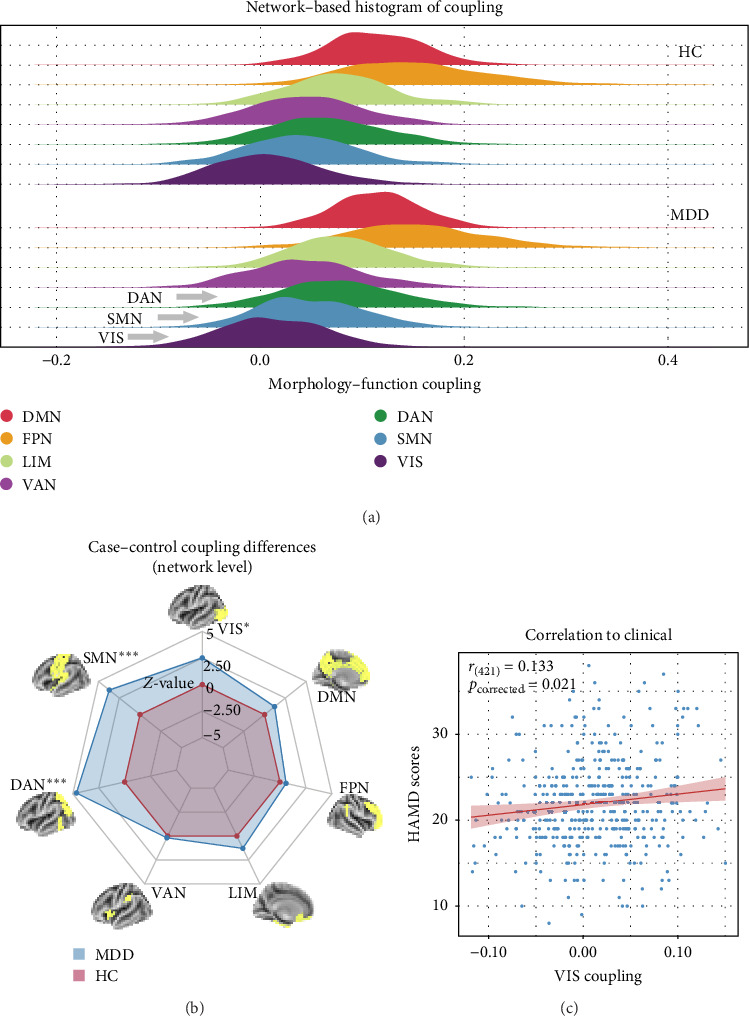
(A) Hierarchical distribution of MFC across the seven cortical networks in the HC group (top) and MDD patients (bottom). (B) Case–control differences in MFC across the seven cortical networks, with MDD patients showing relatively higher MFC in the DAN, SMN, and VIS compared to HC participants. *⁣*^*∗*^*p* < 0.05; *⁣*^*∗∗∗*^*p* < 0.001, BH-FDR corrected. (C) Correlation between HAMD scores and MFC in the VIS (*r* = 0.133, *p*_corrected_ = 0.021), adjusted for age, gender, and education. The gray band represents the 95% confidence interval. BH-FDR, Benjamini–Hochberg false discovery rate; DAN, dorsal attention network; DMN, default mode network; FPN, frontoparietal network; HAMD, 17- item Hamilton Depression Rating Scale; HC, healthy control; LIM, limbic network; MDD, major depressive disorder; SMN, sensorimotor network; VAN, ventral attention network; VIS, visual network.

The results presented in Figures 2 and 3 provide evidence for the intricate coupling relationships between cortical morphological and functional networks. As depicted in Figures 2A and 3A, both MDD patients and HC exhibited a similar spatial organization of MFC. Coupling was stronger in the prefrontal and frontal regions, while it was weaker in primary sensory and temporal cortices. According to Yeo's seven functional systems [37], further analysis revealed that the DMN and the frontoparietal network (FPN) demonstrated the strongest MFC in both groups, while brain networks involved in visual network (VIS) processing and sensorimotor network (SMN) functions showed weaker coupling strength. The global histogram analysis presented in Figure 2B further supported the overall similarity in MFC distribution between MDD and HC, as confirmed by the Kolmogorov–Smirnov test. However, a closer inspection revealed a subtle rightward shift in the MDD distribution, indicating slight yet meaningful differences in MFC patterns despite the overall similarity.

Figure 2C presents a statistically significant negative correlation (*r* = −0.433, *p*_spin_ < 0.001) between the case–control *z*-map and the global MFC of the HC group. This suggested that regions with greater MFC differences between MDD patients and HC correspond to regions with lower MFC in HC participants. Figure 2D reveals a significant increase in global MFC in MDD patients relative to the HC group, as verified by the Mann–Whitney *U* test. In Figure 3B, MDD patients displayed a notable deviation from the controls, with significantly increased MFC in the VIS, SMN, and dorsal attention network (DAN). To control for potential confounds, a partial correlation analysis, adjusting for age, gender, and education, found a statistically significant positive correlation between the changes of MFC in the VIS and HAMD scores among MDD patients (*r* = 0.133, *p*_corrected_ = 0.021, corrected using the false discovery rate method; Figure 3C).

### 3.2. Alterations of Regional MFC in MDD and Their Associations With Meta-Analytic Cognitive Terms Derived From Neurosynth

**Figure 4:**
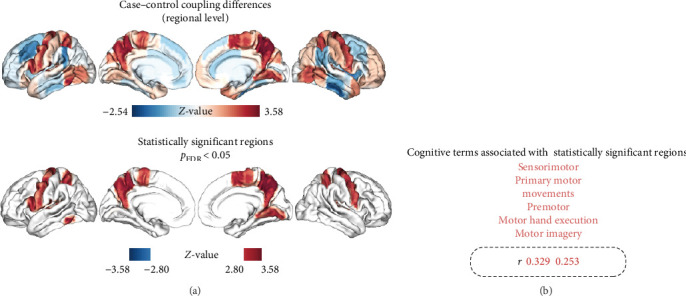
Increased regional MFC and associations with meta-analytic cognitive terms. (A) Regional MFC differences between the MDD and HC groups. (B) Cognitive terms linked to brain regions with significant MFC differences. The font size of each term reflects the correlation coefficient (*r*) of the between-group *z*-map of MFC and the meta-analytic map derived from Neurosynth. The boxes below the word clouds provide a visual representation of the relationship between font size and the correlation coefficient. HC, healthy control; MDD, major depressive disorder; MFC, morphology–function coupling.

As shown in Figure 4A and detailed in Table 2, MDD patients exhibited a significant increase in the mean MFC across multiple brain regions compared to HC. These regions included the bilateral precentral gyri, bilateral superior parietal lobules, bilateral precunei, bilateral planum temporale, left inferior frontal gyrus, left inferior temporal gyrus, left central opercular cortex, right juxtapositional lobule cortex, and right lingual gyrus. Notably, the regions exhibiting increased MFC in MDD were significantly associated with a broad range of cognitive constructs identified through meta-analyses. These constructs were primarily related to motor and executive functions, including sensorimotor integration, premotor activation, motor imagery, and motor execution (as illustrated in Figure 4B).

## 4. Discussion

In this study, we investigated the coupling between cortical morphology and functional networks across the cerebral cortex. Our findings indicate that both MDD patients and HC participants exhibit a similar spatial organization of MFC, with stronger coupling observed in the DMN and FPN, while networks such as the VIS and SMN show weaker coupling. However, the MDD cohort displayed a significant deviation, with a significant increase in MFC across the VIS, SMN, and DAN compared to the control group. In particular, the increase in MFC for the VIS was positively correlated with the HAMD scores. These results point to a disease-specific reorganization of the morphology–function relationship, potentially indicating compensatory mechanisms or pathological alterations associated with MDD.

Structural connectivity in our research is primarily defined by similarities in the cortical GM morphology rather than by the WM tracts typically inferred from diffusion-weighted imaging. This challenges traditional viewpoints and introduces a new perspective on the interplay between brain structure and function. While diffusion-weighted imaging has provided valuable insights into WM connectivity [39], it may overlook the varying patterns of morphological similarity that underlie functional connectivity. Emphasizing these morphological similarities revealed the crucial role of cortical organization in the formation of neural circuits, suggesting that the underlying structural features of cortical regions, such as geometric shape, layering, and neuronal composition, which are key determinants of the strength and patterns of structural connectivity [40]. Thus, investigating how these morphological features influence the functional integration of cortical networks could offer new insights into the mechanisms of the brain structure–function relationship and its abnormalities in psychiatric disorders.

Our results demonstrated a shared hierarchical organization of MFC in both MDD patients and HC, with peak coupling observed in the DMN and FPN. The DMN is known for its role in self-referential processes such as rumination, while the FPN, which is involved in cognitive control and attention, is both vital for adaptive behavior and mental health [41, 42]. This suggests that the structural morphology in these regions corresponds to the brain's resources dedicated to core cognitive and emotional functions. In contrast, the weaker MFC observed in the VIS and SMN in both groups may indicate a generally lower level of dynamic interaction between cortical structure and function in nontask-related networks during resting states. Differences in MFC across functional and neuroanatomical axes highlight variations in functional demands and computational properties across different regions. These findings align with the concept of hierarchical organization in brain function, where primary sensory and motor areas exhibit weaker MFC, reflecting their specialization for rapid and efficient processing of sensory stimuli and motor execution. In contrast, associative regions, such as the DMN and FPN, support higher-level functions, including abstract thinking, social cognition, and cognitive control, which require more extensive interregional integration and adaptability. The differences in the correspondence levels between primary and associative cortices reflect the evolutionary progression of the brain from basic sensory processing to more complex and integrated cognitive functions. These findings underscore the need for a more nuanced approach to understanding the structural and functional relationships in the human brain.

The significant deviations in MFC within the VIS, SMN, and DAN in MDD suggest potential compensatory mechanisms or pathological reorganization in response to the disease state. Previous studies have documented abnormal changes in functional connectivity within these networks in MDD [24, 43, 44]. Our findings are consistent with these results, suggesting that depression is linked to impaired cross-network dynamics and reduced functional flexibility [45]. The increased MFC in the VIS and SMN may represent an attempt by the brain to compensate for functional deficits associated with sensory processing, motor control, or attentional regulation. The involvement of the DAN, which is crucial for directing attention to salient stimuli [46], further supports the hypothesis of compensatory mechanisms. The positive correlation between increased MFC in the VIS and HAMD scores emphasizes the clinical relevance of these alterations, suggesting that they may serve as biomarkers for disease severity or treatment response. Although significant MFC alterations were observed in SMN and DAN, neither showed a significant correlation with HAMD scores (*p* > 0.05). Nonetheless, these findings still hold important clinical implications. The SMN and DAN are primarily involved in sensorimotor processing and top-down attentional control, respectively. While these functions are often impaired in MDD, their clinical manifestations can be highly variable, and the HAMD may fail to fully capture the cognitive and motor symptoms influenced by these networks. Furthermore, functional and morphological changes in SMN and DAN could reflect compensatory or adaptive mechanisms rather than direct pathological disruptions. Alternatively, these networks may be more sensitive to state-dependent factors, such as medication status, duration of illness, or acute stress, which was not explicitly controlled in the present study. Our findings raise questions about the underlying mechanisms driving these alterations in MFC. One possibility is that MDD is associated with neuroplastic changes at the synaptic, cellular, or even structural levels, which underlie the MFC of cortical regions. Another potential explanation could involve changes in neurotransmitter systems, particularly those involved in emotion regulation and stress responses, which may modulate the relationship between cortical morphology and function. Future studies could aim to clarify the causal direction of MFC changes, investigate the temporal dynamics of MFC in MDD, and explore potential therapeutic interventions that target specific alterations in cortical morphology–function relationships.

MDD patients demonstrated a notable increase in regional MFC compared to HC, with pronounced elevations observed in the precentral gyrus, superior parietal lobule, precuneus, planum temporale, as well as the left inferior frontal gyrus, left inferior temporal gyrus, left central opercular cortex, right juxtapositional lobule (supplementary motor area, SMA), and right lingual gyrus. The precentral gyrus, comprising the primary motor cortex, plays a pivotal role in motor and somatosensory functions, as well as threat regulation [47]. The increased MFC in this area may suggest an exaggerated response to perceived threats, which could be a contributing factor to the heightened anxiety and fear often experienced by MDD patients. The superior parietal lobule, which plays a key role in integrating sensory information and directing spatial attention [48], disruptions in MFC may contribute to the attention and perceptual processing deficits frequently observed in MDD [49]. Abnormal MFC in the precuneus, part of the DMN, may be linked to negative self-assessment [50], memory biases [51], and visuospatial orientation issues [52]. These abnormalities mirror symptoms such as impaired memory and distorted self-perception commonly reported by MDD patients [53, 54]. Within the temporal lobes, including the left inferior temporal gyrus, MFC anomalies may provide an explanation for the speech and verbal comprehension impairments observed in MDD [55–57]. These impairments may further exacerbate social isolation and depressive symptoms, contributing to the overall severity of the disorder. The disruptions of MFC in regions vital for semantic operations and sensorimotor functions, such as the left inferior frontal gyrus and the left central opercular cortex [58, 59], have significant impacts on cognitive functions. Specifically, these disruptions affected language and vision-related functions, thereby adversely affecting social functioning. The right juxtapositional lobule is closely associated with other regions involved in motor control and is essential for motor planning and execution [60]. Abnormalities of MFC in this area may reflect difficulties in performing daily tasks, including motor slowing, which is a common symptom of MDD [61]. Finally, the lingual gyrus, associated with visual processing [62], image memory encoding [63], and emotional control [64], exhibited over-coupling indicative of reduced cognitive flexibility and memory impairment, further contributing to the cognitive difficulties experienced by MDD patients. These findings collectively suggest that MDD patients exhibit abnormal MFC across multiple brain regions, reflecting heterogeneous brain area anomalies that may jointly contribute to the pathological processes of the disorder.

Several limitations of this study should be considered. First, while the relatively large sample size enhances the generalizability of our findings, potential heterogeneity within the MDD group remains a concern. Specifically, our dataset does not distinguish between treated and untreated MDD patients, and ongoing treatment may influence brain morphology as well as functional connectivity. Additionally, variations in disease subtypes and comorbidities could also contribute to differences in MFC. To ensure sufficient sample size and statistical power, we included all MDD patients in our analysis rather than restricting the dataset based on treatment status. Thus, future studies incorporating more detailed clinical stratification, including treatment history and medication effects, may provide deeper insights into MFC alterations across different MDD profiles. Second, due to the complex and folded structure of the human cerebral cortex, different morphological indicators can lead to significant variations in regional similarity and network measurements [27, 65]. However, our study focused on a single morphological metric, which may not fully capture the multidimensional nature of MFC. Future studies could integrate multiple morphological features to offer a more comprehensive characterization of cortical structural connectivity. Third, while we used resting-state functional connectivity to assess MFC, it is important to note that brain functional connectivity is dynamic and fluctuates over time [66]. Therefore, further investigation of dynamic MFC in MDD patients is essential to determine whether specific spatiotemporal patterns or fluctuations emerge in the brain under different task conditions or mental states, which would help refine neuroimaging biomarkers for the disorder.

## 5. Conclusion

In conclusion, this study has provided a detailed delineation of the coupling relationships between cortical morphology and functional networks in MDD and unveiled both shared and disease-specific patterns of coupling that contribute to the understanding of the neural substrate of MDD. Specifically, this study highlighted an abnormal pattern of excessive coupling that was intricately linked to cognitive dysfunction and the deterioration of the disorder. Notably, brain regions exhibiting significantly enhanced coupling are associated with the visual, sensorimotor, and executive functions, suggesting a disruption in the typical balance of neural interactions within these critical networks. Furthermore, the observed over-coupling within the VIS indicates a predictive relationship with clinical symptoms, adding another layer of complexity to the understanding of the pathophysiology of MDD. These revelations further enrich our insights into the neural mechanisms underlying MDD.

## Figures and Tables

**Table 1:** Demographic characteristics of the participants.

Characteristics	MDD (*n* = 830)	HC (*n* = 853)	Group comparisons
	(Mean ± SD)	(Mean ± SD)	
Age, years	35.760 ± 12.409	35.766 ± 13.812	*t* = −0.008, *p* = 0.993^b^
Sex, male/female	299/531	344/509	*χ* ^2^ = 3.301, *p* = 0.069^a^
Education level, years	11.739 ± 3.425	13.271 ± 3.442	*t* = −9.152, *p* < 0.001^b^
Mean FD	0.132 ± 0.084	0.135 ± 0.085	*t* = −0.710, *p* = 0.478^b^
HAMD scores	21.957 ± 5.833	—	—
Duration of illness, months^c^	39.055 ± 63.233	—	—

Abbreviations: FD, framewise displacement; HAMD, 17-item Hamilton Depression Rating Scale; HC, healthy control; MDD, major depressive disorder; SD, standard deviation.

^a^The *p*-value was obtained by a chi-square test.

^b^The *p*-value was obtained by a two-tailed two-sample *t*-test.

^c^Data on the duration of illness were available for 830 patients.

**Table 2:** Regions with the significant difference of regional MFC in brain network between participants with MDD and HC group.

ROI	Hemisphere	Coordinate of peak value (mm)			*p*-Value	FDR	*z*-Value
		*x*	*y*	*z*			
Inferior frontal gyrus, pars opercularis	L	−51	14	15	0.004	0.037	2.870
Precentral gyrus	L	−34	−12	49	0.004	0.037	2.895
Precentral gyrus	R	35	−11	50	0.005	0.037	2.836
Inferior temporal gyrus, temporooccipital part	L	−52	−54	−17	＜0.001	0.022	3.575
Superior parietal lobule	L	−29	−50	57	0.003	0.037	2.927
Superior parietal lobule	R	29	−48	59	0.006	0.047	2.729
Juxtapositional lobule cortex (formerly supplementary motor cortex)	R	6	−3	58	0.002	0.032	3.048
Precuneous cortex	L	−8	−60	37	0.001	0.025	3.262
Precuneous cortex	R	9	−59	39	＜0.001	0.022	3.509
Lingual gyrus	R	14	−64	−5	0.004	0.037	2.854
Central opercular cortex	L	−48	−9	12	0.002	0.025	3.170
Planum temporale	L	−52	−30	11	0.002	0.025	3.166
Planum temporale	R	55	−25	12	0.002	0.025	3.167

## Data Availability

The participant dataset is accessible upon reasonable request to the Rest-meta-MDD consortium (http://rfmri.org/REST-meta-MDD).
